# Systemic Adaptions to Extreme Caloric Restrictions of Different Durations in Humans

**DOI:** 10.1038/s42255-024-01008-9

**Published:** 2024-03-01

**Authors:** Maik Pietzner, Burulça Uluvar, Kristoffer J. Kolnes, Per B. Jeppesen, S. Victoria Frivold, Øyvind Skattebo, Egil I. Johansen, Bjørn S. Skålhegg, Jørgen F. P. Wojtaszewski, Anders J. Kolnes, Giles S. H. Yeo, Stephen O’Rahilly, Jørgen Jensen, Claudia Langenberg

**Affiliations:** 1 Computational Medicine, Berlin Institute of Health at Charité – Universitätsmedizin Berlin, Berlin, Germany; 2 Precision Healthcare University Research Institute, Queen Mary University of London, London, UK; 3 MRC Epidemiology Unit, University of Cambridge, Cambridge, UK; 4 Department of Physical Performance, Norwegian School of Sport Sciences, Oslo, Norway; 5 Steno Diabetes Center Odense, Odense University Hospital, Odense, Denmark; 6 Department of Clinical Medicine, Aarhus University, Aarhus, Denmark; 7 Institute of Health and Society, Faculty of Medicine, University of Oslo, Oslo, Norway; 8 Department of Nutrition, Division for Molecular Nutrition, University of Oslo, Oslo, Norway; 9 August Krogh Section for Molecular Physiology, Department of Nutrition, Exercise and Sports, University of Copenhagen, Copenhagen, Denmark; 10 Section of Specialized Endocrinology, Department of Endocrinology, Oslo University Hospital, Oslo, Norway; 11 Metabolic Research Laboratory, Wellcome-MRC Institute of Metabolic Science, University of Cambridge School of Clinical Medicine, Cambridge CB2 0QQ, UK

## Abstract

Surviving long periods without food has shaped human evolution. In ancient and modern societies, prolonged fasting was/is practiced by billions of people globally for religious purposes, used to treat diseases such as epilepsy, and recently gained popularity as weight loss intervention, but we still have a very limited understanding of the systemic adaptions in humans to extreme caloric restriction of different durations. Here, we performed a seven-day water-only fast during which 12 volunteers lost an average 5.7kg (±0.8kg) of weight. We performed in-depth characterisation of the temporal trajectories of ˜3,000 plasma proteins measured before, daily during, and after fasting to demonstrate, for the first time, nine distinct proteomic response profiles, with systemic changes evident only after three days of complete calorie restriction. The multi-organ response to complete caloric restriction showed distinct effects of fasting duration and weight loss and was remarkably conserved across volunteers with >1000 significantly responding proteins. The fasting signature was strongly enriched for extracellular matrix (ECM) proteins from various body sites, demonstrating profound non-metabolic adaptions, including extreme changes in the brain-specific ECM protein tenascin-R. Using proteogenomic approaches, we estimated the health consequences for 212 proteins that changed during fasting across ˜500 outcomes and identified putative beneficial (SWAP70 and rheumatoid arthritis or HYOU1 and heart disease), as well as adverse effects. Our results advance our understanding of prolonged fasting in humans beyond a merely energy-centric adaptions towards a systemic response that can inform targeted therapeutic modulation.

## Introduction

The adaption to prolonged periods without food is one of the greatest evolutionary challenges of humanity, but we know little about how humans adapt to and survive starvation beyond the shift from glucose to fat and ketone body metabolism. With the development of settlements and agriculture, and hence often abundance of food, fasting emerged as a voluntary religious practice of renunciation to achieve purification^1^ and as an ancient medical treatment, including childhood epilepsy^2^ or rheumatoid arthritis^3^. More recently, different intermittent fasting schemes have been proposed for prevention and treatment of obesity, associated cardiometabolic disorders, and neurodegenerative diseases, with an ongoing debate on whether prolonged fasting (≥12 to 48 hours) provides health benefits over and above comparable reductions in calorie intake at each regular meal^3–6^. Large prospective clinical trials that causally link these fasting schemes to health benefits or molecular insights that support such results are, however, largely lacking^6^. An enhanced understanding of the systemic adaptions to prolonged fasting in humans will not only identify new metabolic regulators but also help to explain the proposed benefit of fasting-mimicking diets, e.g., ketogenic diets, as adjunct to chemotherapy for colorectal^7^ or breast cancer^8,9^.

Broad-capture proteomic techniques can now measure thousands of plasma proteins simultaneously with high specificity at scale and thereby provide the opportunity to systematically, and agnostically, study molecular adaptions to fasting in humans in unprecedented detail. This could complement and address limitations of earlier studies of selected candidates, like insulin-like growth factor 1 (IGF-1) or adiponectin^3,5^, or small molecule profiling^10^. Further, we^11,12^ and others^13–15^ have recently demonstrated how methodological approaches that integrate human proteomic and genomic data can identify novel pathways to diseases. This uniquely enabled us to combine understanding from proteomic changes driven by a standardised extreme caloric restriction intervention with causal understanding of the links between those same proteins with hundreds of diseases through independent, large-scale genomic studies.

Here, we profiled plasma proteomic trajectories before, during and after a seven-day water-only fast among twelve generally healthy young volunteers and generated proteogenomic evidence of possible health consequences. We observed a homogeneous, multi-organ response across volunteers affecting a third of the plasma proteome that segregated into different temporal clusters of responding pathways and organ systems, with profound systemic changes not occurring until after three days of fasting. Our results corroborate observations from trials that show limited evidence for beneficial effects of intermittent fasting (≤48 hours) over and above matched caloric restriction^6,16–18^. We provide selected examples where our results provide potential insights into mechanisms underlying the health benefits of fasting on disease states, including some, like rheumatoid arthritis, where the benefits are well documented and others, like cardiovascular disease, where they are more speculative. Our findings have the potential to derive tailored therapeutic options for patient groups for whom fasting or ketogenic diets are contra-indicated, and/or or circumvent the challenges to adherence to these types of diet.

## Results

### Study design

**Figure 1:**
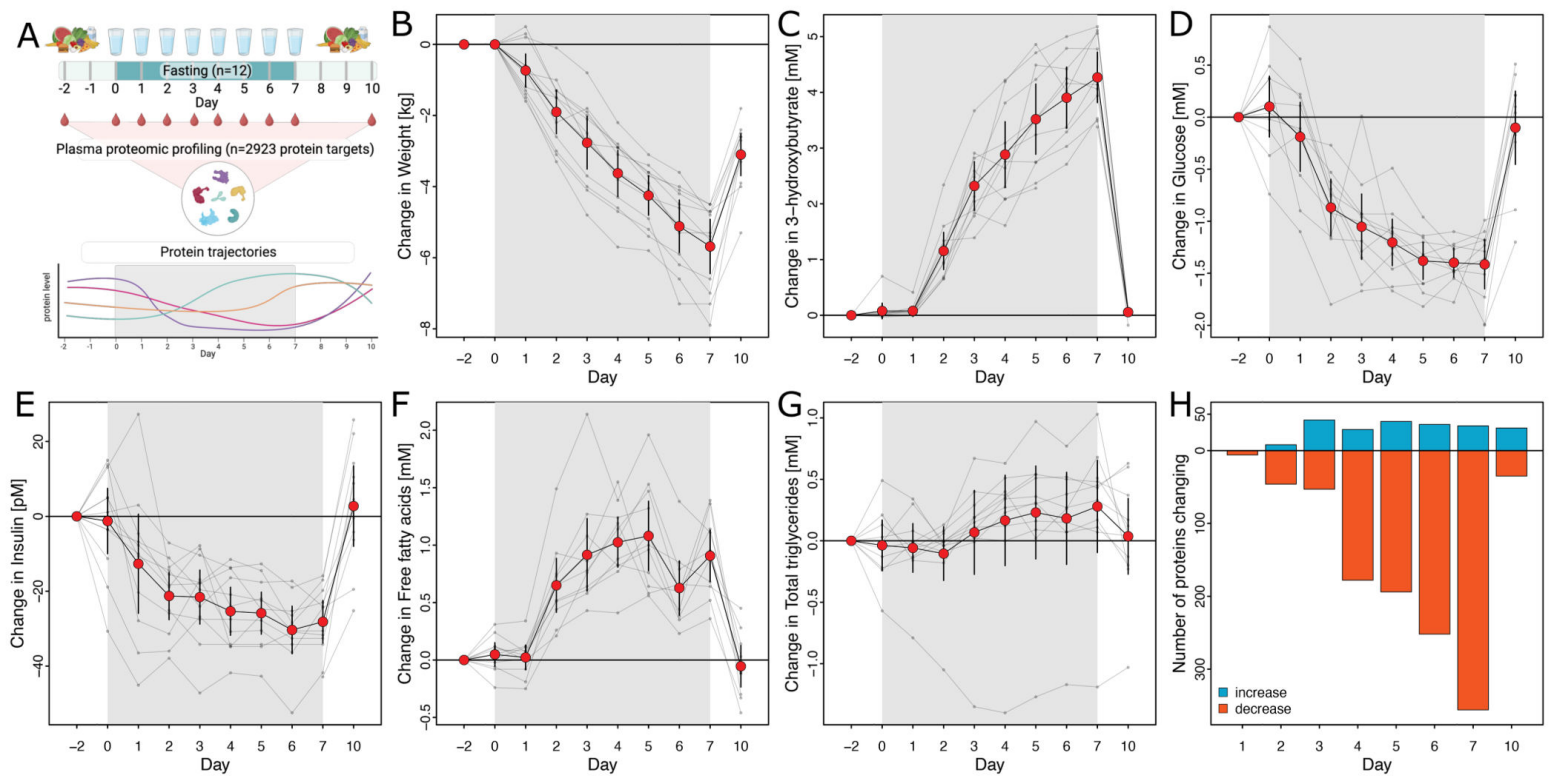
Study design and core participant characteristics. **A** Sketch of the study design **B-G** Change (mean ± SEM) in each of six participant characteristics and blood parameters in original units. Thin lines indicate changes in individual participants. Corresponding association statistics and absolute values can be found in Supplementary Tab. 1. **C** Summary of proteins with significantly different plasma levels (p<4.7x10^-5^) compared to baseline.

We studied twelve participants (5 women and 7 men; Supplemental Tab. 1) before, during and after a prolonged 7-day fast with absolute caloric restriction and *ad libitum* water intake only. We sampled fasting blood early in the morning (6-9am) from all participants two days prior the fasting intervention, daily during the intervention, and three days after stopping the intervention (Fig. 1). We measured plasma levels of a total of 2923 protein targets using 2941 assays in 117 samples based on the Olink 3072 Explore platform (see Methods).

At the beginning of the study, participants weight on average 77.5kg (s.d.: 15.7kg) and had an average body mass index (BMI) of 25.4kg/m^2^ (s.d.: 4.1 kg/m^2^). Over seven days of fasting, participants lost an average 5.7kg (SEM: ±0.8kg) of weight, equivalent to 1.9 units (±0.18) of BMI, weight remained below the initial body weight (average: -3.1 ± 0.6kg) over the next three days of *ad libitum* food intake (Fig. 1). Weight loss was attributable to both, total lean (-3.6 ± 0.49kg; p-value<5.5x10^-12^) and fat mass (-1.6 ± 1.3kg; p-value<9.9x10^-13^). Fat mass decreased significantly in the subcutaneous (-0.21 ± 0.07kg; p-value<5.1x10^-8^) but not visceral fat (-0.07 ± 0.09 kg; p=0.12) compartment, and no significant loss of bone mass (0.008 ± 0.014 kg; p=0.42) was observed. The loss of lean mass was almost completely reversed (-0.69 ± 0.49kg) during the three days following the end of fasting while the loss in fat mass (-1.85 ± 0.34kg) was sustained (Supplemental Fig. 1).

We observed the expected switch from glucose to lipid utilization within the first two to three days, with plasma glucose concentrations dropping while fatty acid concentrations increased and plateaued thereafter (Fig. 1). Results resembling previous studies^19,20^. A continuous rise in plasma 3-hydroxybutyrate concentrations throughout the entire fasting period provided evidence of increased ketogenesis to meet energy demands (Fig. 1). Participants did not report any severe adverse events during daily interviews, and we ensured high adherence through daily contact with participants also supported by the described metabolic adaptions resembling well-described changes during fasting. Urinary nitrogen levels decreased only late during the fasting study period, likely due to increased renal reabsorption of urea to spare protein pools^20^ (Supplementary Tab. 1 and Supplementary Fig. 2).

### Prolonged fasting has systemic effects on the human plasma proteome

One third of all protein targets (35.9%, n=1044) changed significantly during fasting (false discovery rate (q-value) <5%; see Methods; Supplementary Tab. 2), with 144 protein targets increasing (n=22) or decreasing (n=122) by more than 2 s.d. units during the study period. A systemic plasma proteomic response was only evident after three days of fasting, with few significant (p<4.7x10^-5^) proteins changing after 24 (n=6) or 48 hours (n=54) (Fig. 1H). Most proteins decreased during the study period, the number of which grew exponentially after several days of fasting while the number of proteins that increased remained rather stable following day 3 (Fig. 1H). Three days after participants resumed eating, 66 proteins were still significantly different from pre-fasting plasma levels.

We observed the expected reduction in plasma leptin levels^21^ (max at day 4: 2.39 s.d. units decrease, p<7.7x10^-34^) and suppression of the hypothalamus-pituitary-thyroid axis (thyrotropin: min at day 2: 1.41 s.d. units decrease). Other proteins that have been proposed to be important mediators of the response to feeding/fasting showed insignificant or only modest changes. These included ghrelin (min at day 1: 0.28 s.d. units increase, p=0.66), adiponectin (min at day 7: 0.46 s.d. units decrease, p=0.01) and brain-derived neurotrophic factor (min at day 7: -0.47 s.d. units decrease, p=0.57) (Supplementary Fig. 3). In contrast, proteins like follistatin (FST, max at day 3: 5.62 s.d. units increase, p<3.2x10^-44^), proprotein convertase subtilisin/kexin type 9 (PCSK9, min at day 7: 4.75 s.d. units increase, p<4.1x10^-47^), or neuroblastoma suppressor of tumorigenicity 1 (NBL1, min at day 7: 5.20 s.d. units decrease, p<8.4x10^-15^), markedly changed (>4 s.d. units) and may represent novel markers of prolonged fasting (≥3 days) (Supplementary Fig. 4). Only three proteins showed evidence of a significant sex-differential effect (Supplementary Fig. 5).

On a pathway level, we observed well-defined fasting-induced changes in insulin-like growth factor (IGF) signalling, cytokine signalling, lipoprotein metabolism and metabolism of proteins more generally, to be enriched among all significantly altered proteins. This also included less described changes in the complement and coagulation cascade, altered protein glycosylation, cell adhesion, or neutrophil degranulation (Supplementary Fig. 6).

**Figure 2:**
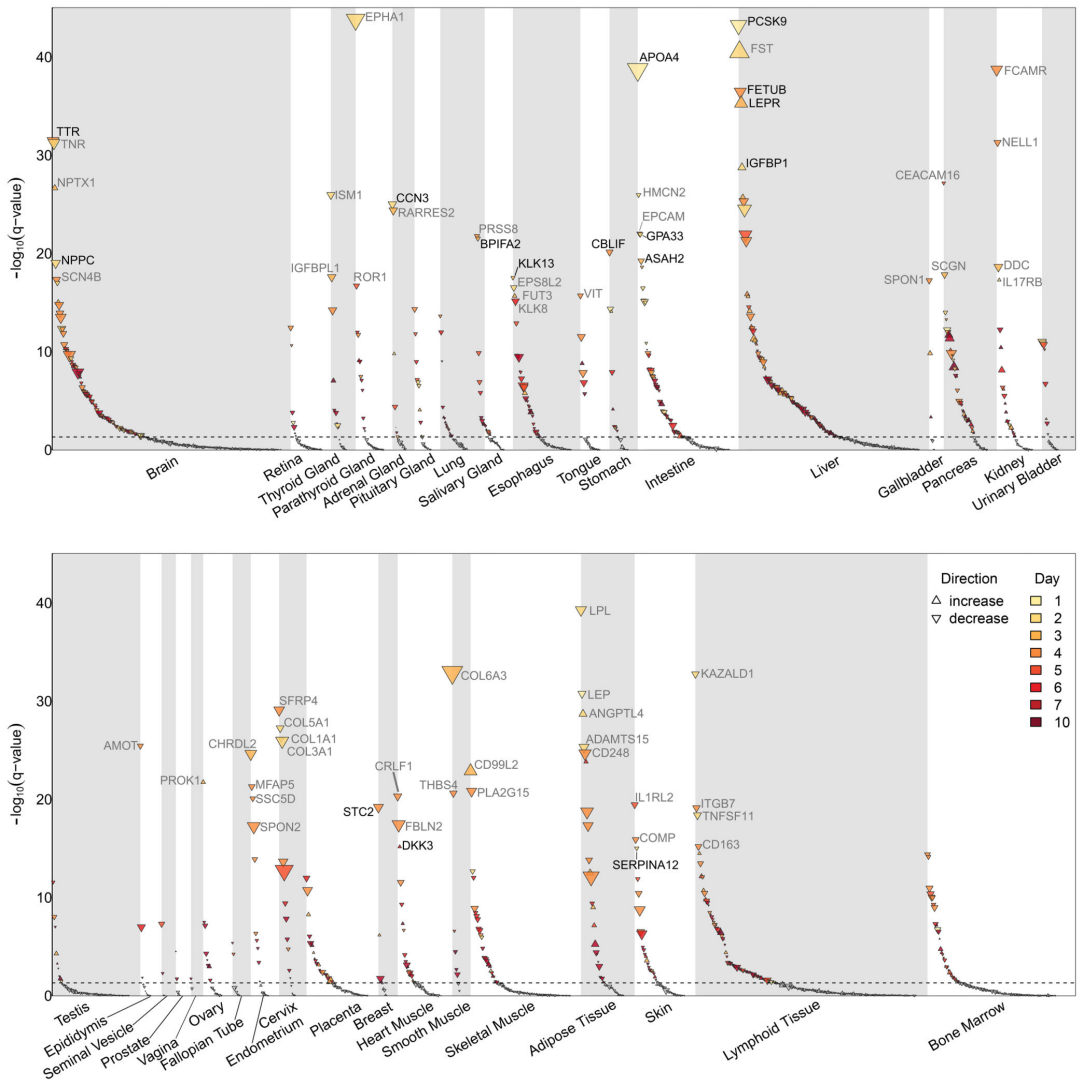
Significantly associated plasma proteins with evidence of tissue enrichment. Adjusted p-values, false-discovery rate (q-value), from mixed effect linear regression models for a time effect across 1837 unique protein targets with evidence for tissue enrichment in the Human Protein Atlas. Proteins are grouped based on the tissue with the highest gene expression according to the Human Protein Atlas^22^. The size of the dots is proportional to the absolute maximal amplitude during the study, and the colour of the dots indicates the day when the respective protein first showed statistically significant (p<4.7x10^-5^) differences compared to baseline levels. Top five proteins in each tissue are labelled if their –log10(q-value) was above 15. Figure is split into two rows to fit into the page, and tissues are ordered anatomically. The underlying data can be found in Supplementary Tab. 2.

Proteins in plasma have diverse origins, and we found that many proteins that changed during fasting were mainly, or even exclusively, expressed in specific tissues spanning all major organ systems recorded in the Human Protein Atlas^22^ (Fig. 2), with a significant enrichment of proteins that are specifically expressed in liver, pancreas, adipose tissue, and intestine (Supplementary Fig. 7). Single-cell RNA sequencing data refined exocrine glandular cells, hepatocytes, smooth muscle cells, intestinal goblet or collecting duct cells as likely relevant subpopulations in each of those tissue along with revealing less obvious contributions from fibroblasts and thymic epithelial cells (Supplementary Fig. 8).

### Distinct trajectories of proteomic changes

**Figure 3:**
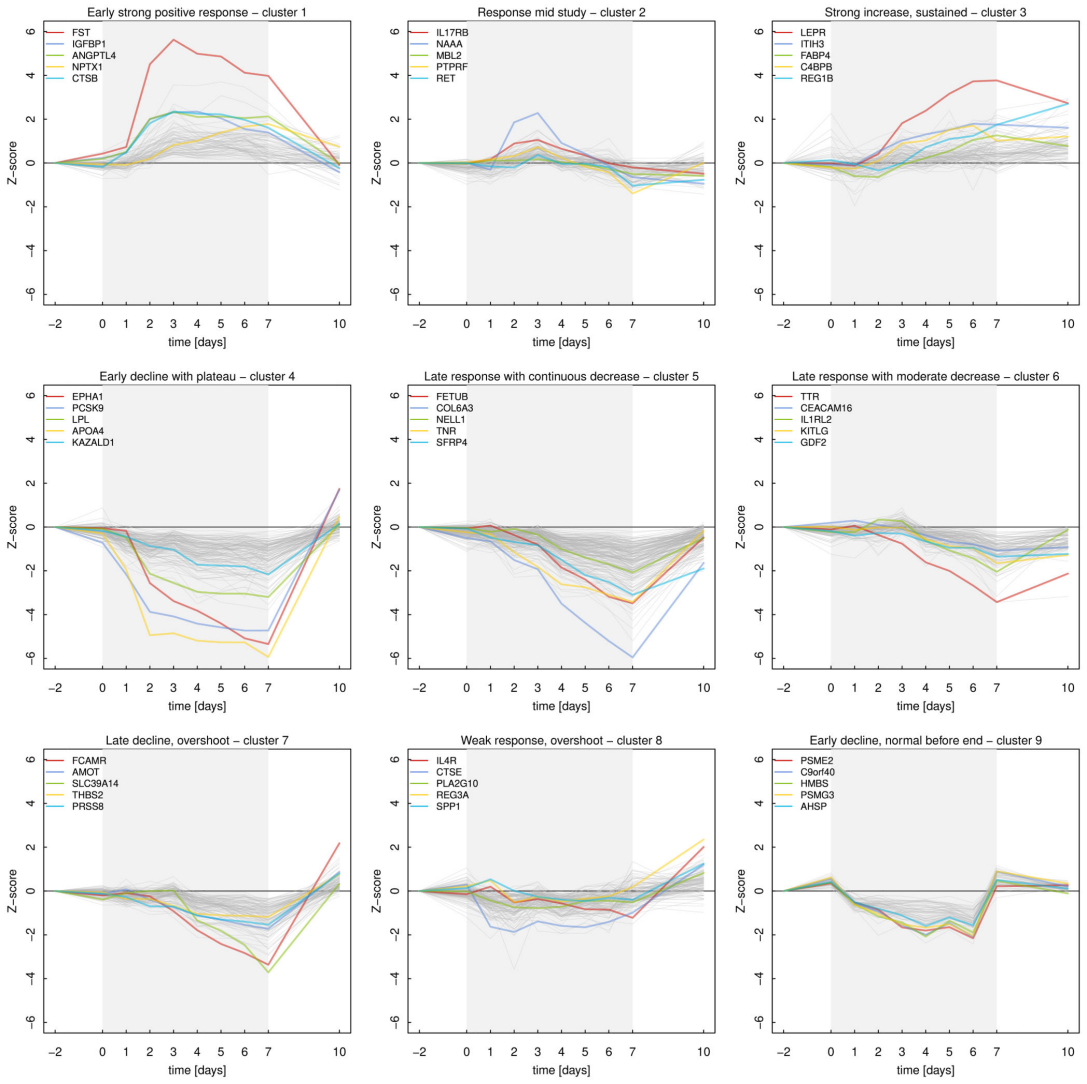
Temporal trajectories of plasma protein levels during fasting. A total of 1034 protein targets that showed significant changes during the study course (q-value<0.05) distributed across nine temporal clusters. Each panel shows standardized mean values (based on mean and s.d. from two days before fasting) of all protein targets belonging to the respective cluster. The five protein targets most significantly changed (based on q-value) are highlighted by colours. Fasting started on day 0 and ended on day 7. The underlying statistics can be found in Supplementary Tab. 2.

We identified nine clusters among 1,034 significantly changing protein targets that reflected distinct dynamic adaptions to sustained fasting and rebound, broadly segregating into proteins that increased (3 clusters) or decreased (6 clusters) along different temporal profiles during the study (Fig. 3). The three clusters containing increasing proteins (cluster 1-3) indicated adaptions to sustain hunger signalling, reduce reproductive function, decrease LDL-cholesterol uptake by the liver (Supplementary Fig. 9), increase protein catabolism, promote a putatively hypercoagulable state, transient inflammation, and a potential compensatory response of the exocrine pancreas and adipocyte function at the end of the study. Proteins that showed an early and sustained increase (cluster 1) included signalling peptides like agouti-related peptide, key regulators of lipid metabolism (LDL-receptor and ANGPTL4), IGF binding proteins, and were further enriched for ‘factor IX activation’ (Supplementary Fig. 6) and proteins involved in protein degradation (e.g., cathepsins B and L). Proteins that were transiently increased at day two and three indicated moderate activation of the complement cascade (p<1.6x10^-5^), while proteins persistently elevated, even following food intake, included those secreted by the exocrine pancreas (e.g., REG1B or REG3G) or were related to adipocyte biology (e.g., fatty acid binding protein 4).

The latter was further reflected in proteins that declined and plateaued thereafter within two to three days (cluster 4; Fig. 3). These proteins were indicative of the metabolic switch from glucose towards peripheral lipid release and utilization, as well as intracellular uptake or diminished recycling of extracellular matrix (ECM) proteins to spare protein pools during prolonged fasting^23^. This included proteins involved in appetite regulation (PYY), lipoprotein turnover and lipid metabolism (e.g., PCSK9, lipoprotein lipase [LPL], or apolipoprotein A-IV [APOA4]), ECM organization (e.g., collagens or hyaluronidase 1), iron metabolism (e.g., hemojuvelin BMP co-receptor), regulators of insulin secretion and glucose metabolism (e.g., kirre like nephrin family adhesion molecule 2 [KIRRLE2] or fam3 metabolism regulating signalling molecule d [FAM3D]), as well as adipokines, like leptin or isthmin-1^24^. Beyond such general metabolic adaptions, strong (≥2 s.d. units) and sustained changes in proteins like ephrin type-A receptor 1 (EPHA1) or BPI fold containing family A member 2 (BPIFA2) were among the most prominent novel findings that might indicate altered epithelial cell motility^25^ and antibacterial defence in the respiratory system^26^, respectively.

Remarkably, most proteins did not decline before day two or three (clusters 5-7) and pointed to different pathways compared to early changes. For example, proteins enriched for ECM-receptor interactions (p<1.1x10^-8^), molecules associated with elastic fibres (p<4.8x10^-4^), and cell surface interaction at the vascular wall (p<5.1x10^-3^; Supplementary Fig. 5) declined from day two. This may likely reflect structural adaptions to prolonged starvation in the vasculature, including degradation of the ECM that might explain, to some extent, the loss in lean mass. These changes were further paralleled by multiple hormonal signals, including the hepatokine fetuin-B^27^ or the adipokine chemerin (RARRES2), as well as proteins involved in bone metabolism, such as secreted frizzled related protein 4 (SFRP4) and osteoglycin (OGN)^28^. Following day three, a cluster of proteins enriched for members of the complement and coagulation cascade (p<1.4x10^-4^) started to decline (Supplementary Fig. 10). This was accompanied by reductions in thyroid hormone transport (e.g., transthyretin), further progressive loss of proteins of the ECM, and different cytokines and associated receptors. While most of the proteins of these late clusters returned to or, at least, showed a trajectory towards baseline levels, immune cell surface markers, like Fc Alpha and Mu receptor (FCAMR), markers of cellular senescence, like galactosidase beta 1 (GLB1), and proteins involved in angiogenesis, such as angiomotin (AMOT), spondin 1 (SPON1), or thrombospondin 2 (THBS2), overshot pre-fasting levels after participants resumed eating.

We identified a similar and potentially compensatory response at the end of the study for a cluster (cluster 8) enriched for proteins secreted from the exocrine pancreas (p<2.1x10^-3^). This included regenerating islet-derived protein 3 alpha (REG3A) and elastases (e.g., CELA3A), and markers of bone formation, like osteopontin, that changed only weakly, if at all, during the study otherwise.

We finally identified a cluster (cluster 9) enriched for proteins involved in ‘cellular response to stress’ (p<2.5x10^-5^), like proteasome activator subunit 2 (PSME2), and different metabolic pathways (including nucleotide metabolism: p<5.8x10^-3^) that returned to baseline levels already on day 7 before participants resumed eating. The intracellular origin of most of the associated protein targets may indicate increased leakage from, among others, erythroid cells (Supplementary Tab. 2) as a putative result from adaptions in blood cell composition in response to prolonged fasting.

### Differential effects of prolonged fasting versus weight loss

**Figure 4:**
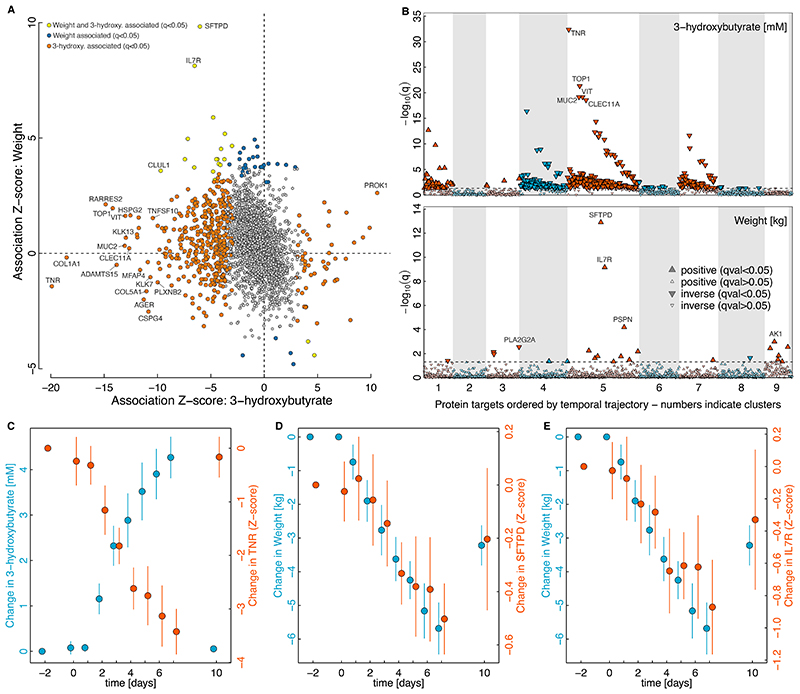
Proteins associated with changes in plasma 3-hydroxybutyrate and weight. **A** Scatterplot opposing Z-scores (beta/s.e.) from linear regression models associating changes in plasma 3-hydroxybutyrate levels (x-axis) and weight (y-axis) with plasma protein levels during the entire study period. Dots coloured in red were significantly associated (false discovery rate < 5%) with both exposures, whereas dots coloured in orange or blue were significantly associated only with either 3-hydroxybutyrate or weight, respectively. **B** The two panels display corrected p-values (q) from mixed effect linear regression models associating changes in plasma 3-hydroxybutyrate levels and weight with identified plasma protein clusters ordered by temporal changes (see Fig. 2) and by strength of association within each cluster. Parameter – protein associations passing multiple testing (q<0.05) are highlighted by larger symbols and darker colours. In each panel the ten most significant proteins have been annotated. Positive associations are indicated by upward facing triangles, whereas inverse associations are indicated by downwards facing triangles. Note that positive associations with weight imply lower protein levels during weight loss. **C-D** Mean ± SEM for significantly associated participant characteristics and corresponding protein levels from mixed effect linear regression models. Blue indicates participant characteristics, whereas orange indicates the protein level.

Controversy exists whether fasting of at least twelve hours or longer during different times of the day or week (intermittent fasting) exerts beneficial effects on health and weight loss over and above comparable caloric restriction, with trials reporting a large variation ranging from moderate to no effects, although very few compared to matched caloric restriction^3–6^. Plasma ketone bodies, in particular 3-hydroxybutyrate, that strongly rise under prolonged fasting, are thereby considered as effector molecules based on putative central and peripheral signalling capabilities^29^. We found that plasma proteomic profiles of changes in weight during the study were only weakly correlated (r=-0.20; Fig. 4A) with changes in plasma 3-hydroxybutyrate levels, with most proteins (n=452; Fig. 4B) being significantly (q-value<0.05) associated with fasting rather than weight change (n=49; Fig. 4B and Supplementary Tab. 3). This observation might imply that prolonged fasting exerts effects distinct from short-term weight loss. However, early changes (≥48h) were subtle compared to the profound changes occurring after three or more days of fasting, a duration longer than all commonly used fasting strategies.

### Prolonged fasting is linked to surrogates of neural ECM organization

The fasting protein signature was strongly enriched for proteins of the ECM (p<3.6x10^-7^), with tenascin-R, a brain-specific ECM protein, being the most significantly associated candidate (beta=-0.73, p<2.4x10^-37^; Fig. 4C). Tenascin-R is integral for the structural maintenance of perineuronal nets that stabilize neurons and synapses and therefore critically determine synaptic plasticity^30,31^. Although perineuronal nets are considered remarkably stable, more recent work indicated constant recycling of tenascin-R by neurons, preferentially near synapses^32^. One might speculate that fasting-induced metabolic reprogramming of neurons also affect tenascin-R recycling and subsequently reduced loss into the circulation. Diets that raise plasma ketone bodies have been used for centuries to treat epilepsy, particularly in children, as an alternative to restrictive fasting schemes and are still an established treatment for drug-resistant epilepsy attributed to a, yet to be defined, effect of ketone bodies on neurons^33^. Our observation, that a rise in plasma 3-hydroxybutyrate is strongly associated with lower plasma levels of likely brain specific members of the ECM (e.g., brevican or vitrin) and neutrophic factors (e.g., neurotrophin 4) provide a potential new path to help understand the success of this ancient treatment scheme.

### The proteomic signature of weight loss is linked to COVID-19 and autoimmune diseases

Pulmonary surfactant-associated protein D (SFTPD; beta=0.08, p<7.0x10^-17^; Fig. 4D) and interleukin 7 receptor (IL7R; beta=0.12, p<9.3x10^-13^; Fig. 4E) stood out among the proteins most strongly associated with weight trajectories during fasting. Both followed weight changes in a consistent manner, declining with weight loss and increasing following weight rebound after participants resumed eating. Both proteins have important roles in immunity; SFTPD participates in the first line response to pathogens in the lung and has recently been linked to critical illness in COVID-19^34^, whereas IL7R is pivotal for T-cell development and malfunctioning has been linked to diseases with an autoimmune aetiology, including multiple sclerosis^35^, and more recently asthma^36^. The consistent associations of both proteins with weight change in the current study may make them attractive candidates for the mediation of adverse events associated with adiposity. For example, adiposity is an established risk factor for severity of COVID-19^37^ and multiple sclerosis^38^, and SFTPD and IL7R may play a role in mediating its adverse effects on these diseases. This hypothesis is supported by our previous work demonstrating that life-long higher plasma levels of both proteins due to common genetic variation at both protein coding genes was associated with an altered risk for both diseases^39,40^. For example, we showed that the same genetic variant that increases plasma levels of IL7R also increased the risk for multiple sclerosis^39^.

### Putative proteomic mediators of the metabolic response to fasting

We next sought to identify proteins possibly causally mediating the metabolic adaptions to prolonged fasting by integrating evidence from the present and human genetic studies^12^. We prioritized a total of 20 protein targets (Fig. 5) that: 1) strongly correlated with blood-based markers of glucose and lipid metabolism in the present study (Fig. 5B), and 2) had strong genetic evidence that DNA sequence variation that influences proteins levels also predisposes to altered glucose and lipid metabolism, or related disorders (Fig. 5A, Supplementary Tab. 3-4). This included established (PCSK9 and ANGPTL4) and emerging (asialoglycoprotein receptor 1 [ASGR1] the classical hepatocyte receptor for desialyated glycoproteins that has recently been suggested to be a ligand for the LDL-receptor^41^) regulators of lipoprotein uptake and metabolism, as well as novel candidates like Inhibin beta C chain (INHBC). The INHBC-increasing G-allele of the single nucleotide polymorphism rs61352607 (beta=0.65; p<3.4x10^-139^) was positively associated with plasma triglyceride levels (beta=0.03, p<7.8x10^-33^; posterior probability [PP] same genetic signal = 96.0%; Supplementary Tab. 4), in line with plasma levels of INHBC being positively associated with plasma triglyceride levels in the present study (beta=0.49, p<3.7x10^-5^). INHBC is specifically expressed in the liver, where it typically dimerises to form activins, in contrast to dimerization of the beta C chain being considered a mechanism of inactivation^42^. Activins act in concert with inhibins to regulate the sex-specific release of gonadal hormones, in particular follicle stimulating hormone and are essential for normal morphogenesis of multiple organs^42^. The possible endocrine role of INHBC for triglyceride metabolism has yet to be elucidated.

### Genetically predicted health impact of prolonged fasting

**Figure 6:**
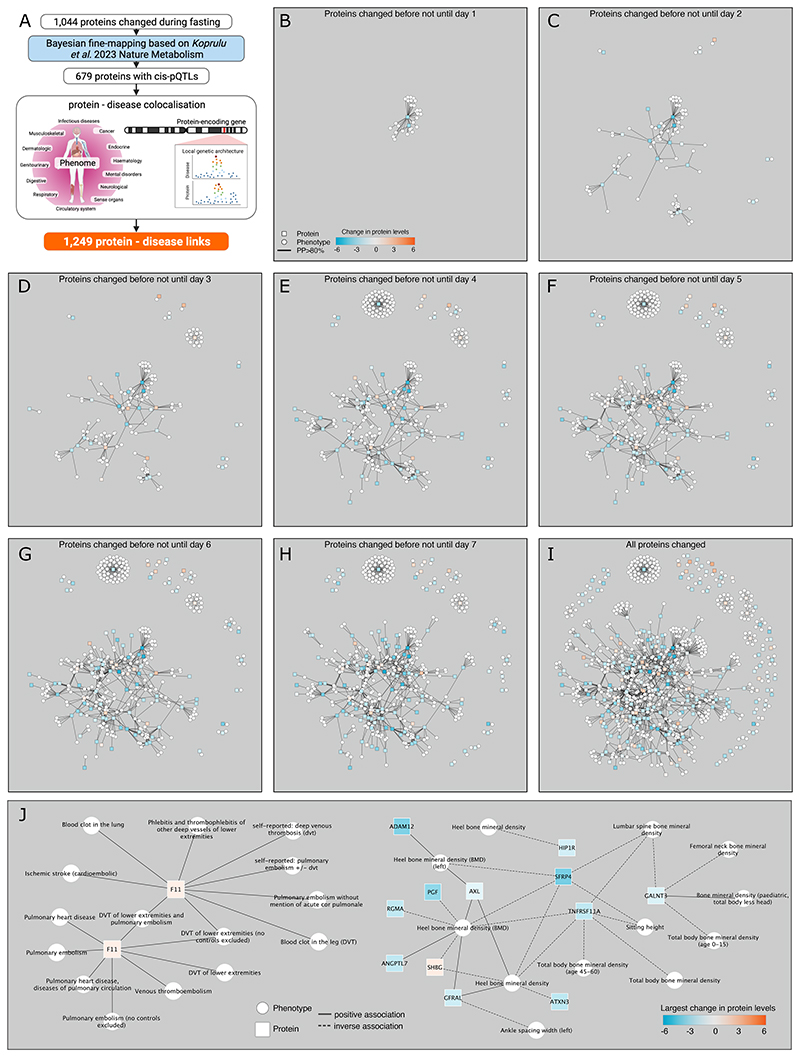
Protein – disease networks. **A** Flow-chart for the identification and linkage of protein quantitative trait loci (pQTL) in close proximity to the protein-encoding gene with diseases and health measures. **B – I** Protein – disease networks in which proteins (white squares) are linked to phenotypes (grey circle) if they had strong evidence (posterior probability >80%) of a shared genetic signal at the protein coding loci. Networks are built up sequentially including only proteins that changed significantly during fasting until the day given in the legend. **J** Sample networks centred around two distinct causal genetic variants for factor XI and proteins putatively causally related to bone phenotypes.

We finally estimated the potential health impacts of the 1,044 proteins significantly changed during fasting by first identifying genetic variants linked to plasma proteins levels, to subsequently systematically establish a shared genetic architecture with diseases and diverse measures of health substantially expanding upon our previous work^12^. We successfully established putatively causal links by which genetic liability towards higher/lower plasma levels of 212 proteins was linked to almost 500 diseases and health measures (Fig. 6 and Supplementary Tab. 4; see Methods). We observed little evidence of an effect of those proteins that significantly (p<4.7x10^-5^) changed early on, i.e., after a short period of fasting of less than 2 days, (Fig. 6B-C) on health outcomes, except for lipid metabolism related proteins like PCSK9, LPL, or ANGPTL4.

In contrast, proteins that changed late and after three or more days of fasting showed diverse effects on multiple health outcomes (Fig. 6D-I). These results overall established a diverse proteogenomic map (Fig. 6D-I), including links to diseases known to benefit from prolonged fasting. For example, we identified a shared genetic signal between plasma levels of switch-associated protein 70 (SWAP70) and rheumatoid arthritis (RA) (PP=99.1%). The A-allele of rs4910499 (minor allele frequency (MAF)=36.9%) associated with higher plasma levels of SWAP70 (beta=0.28; p<4.0x10^-27^) and carried a significantly higher risk of RA (odds ratio: 1.08; p<6.7x10^-10^). The genetic locus has first been identified in Korean populations^43,44^ and was suggested to be involved in the pathology of RA via its role in interferon signalling and B-cell migration. Plasma levels of SWAP70 declined significantly during the study period (p<6.0x10^-5^), peaking at day 6 with an average drop of 1.5 s.d. units compared to baseline. Assuming that SWAP70 is not only linked to RA onset, but also symptoms this might provide at least a partial explanation for the pain relief of RA patients during prolonged fasting^45^.

More generally, we observed that for more than half of the genetically predicted protein – disease links (n=652; 52.2%) prolonged fasting seem to compensate for potentially adverse effects, including complement receptor 1 and CD2 associated protein linked to Alzheimer’s disease (Supplemental Tab. 4). For the remainder of the examples, in contrast, prolonged fasting affected protein levels in a way that aligned with genetically predicted increased disease risk, such as increasing levels of coagulation factor XI that are genetically linked to a higher risk for thrombotic events (Fig. 6J; Supplemental Tab. 4). We also observed evidence for potential beneficial as well as harmful effects on bone health (Fig 6J). Seven proteins for which lower plasma levels were genetically linked to greater bone mineral density declined during the study (TNF receptor superfamily member 11a [TNFRSF11A], secreted frizzled related protein 4 [SFRP4], huntingtin interacting protein 1 related [HIP1R], repulsive guidance molecule A [RGMA], placenta growth factor [PGF], polypeptide N-acetylgalactosaminyltransferase 3 [GALNT3], and ataxin 3 [ATXN3]). In contrast, bone promoting markers like AXL receptor tyrosine kinase (AXL), growth differentiation factor 15 receptor (GFRAL), disintegrin and metalloproteinase domain-containing protein 12 (ADAM12), or angiopoietin-related protein 7 (ANGPTL7) declined as well (Supplemental Tab. 4). These changes in bone-related proteins occurred in the absence of an effect on total bone mass during the study period (p=0.42).

### Novel fasting-modulated regulators of cardiovascular risk

**Figure 7:**
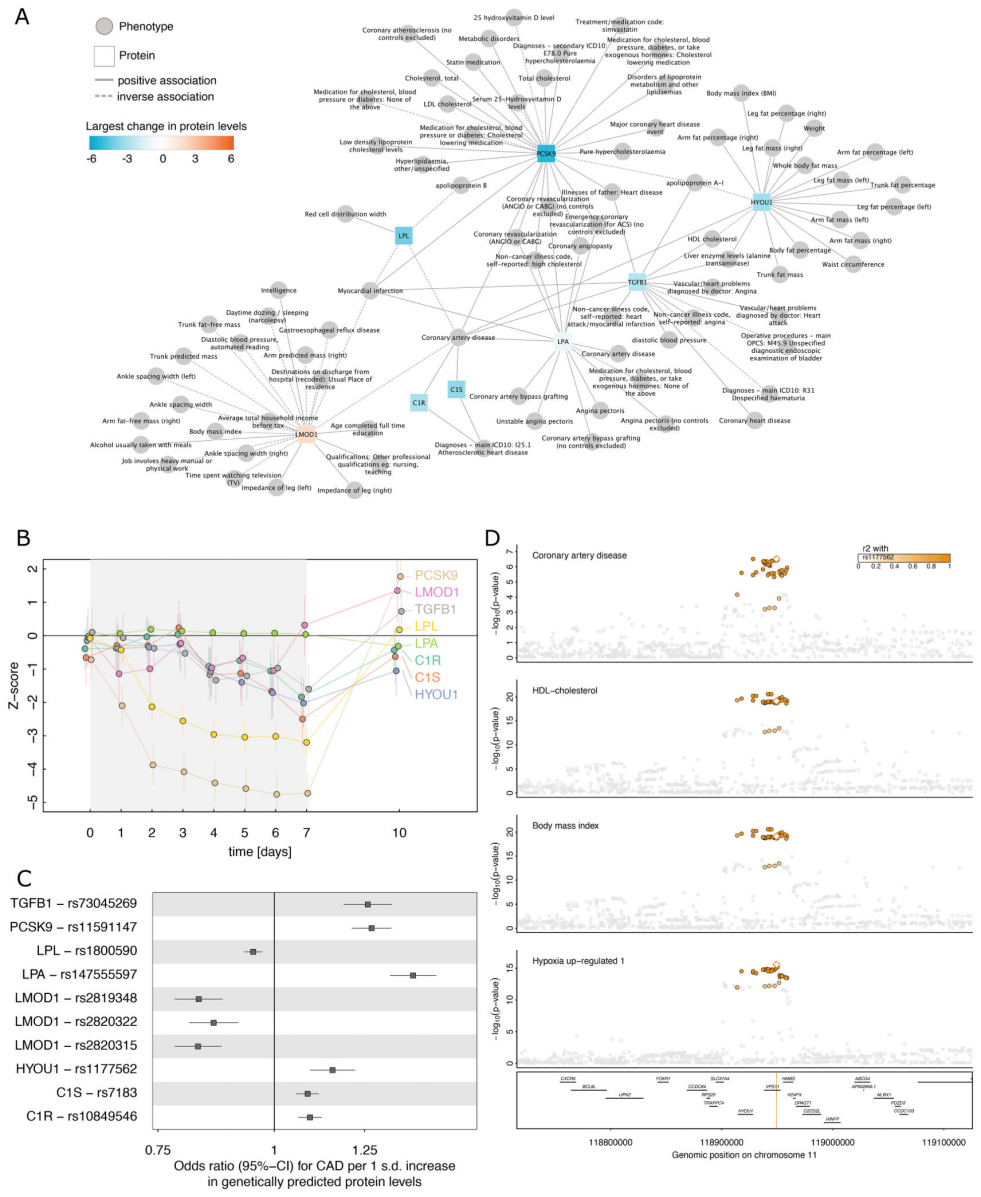
Fasting proteins with a putative causal role in coronary artery disease (CAD). **A** Genetically inferred protein – disease network centred around CAD. Squares indicate proteins and circles diseases and health characteristics. Proteins were connected to phenotypes if both shared the same genetic variant affecting blood protein levels and phenotype characteristics/disease risk based on statistical colocalization (Posterior probability >80%). Solid edges indicate that the protein-increasing allele was associated with higher risk for the phenotype, and dashed lines the opposite. Proteins were coloured based on their largest amplitude during the study period. Note that LMOD1 has been measured by multiple assays and all findings are presented for consistency. **B** Temporal trajectories of target proteins during the study period. Shown are effect estimates and corresponding 95%-confidence intervals from mixed effect linear regression models. Colours indicate respective proteins. **C** Odds ratios and 95%-confidence intervals from estimating the effect of genetic liability to higher protein levels on CAD risk based on the lead genetic variant in the protein encoding locus (±500kB). **D** Regional association plot for the shared genetic signal at *HYOU1* across plasma levels of hypoxia up-regulated 1 (HYOU1), body mass index, high-density lipoprotein (HDL) cholesterol, and CAD. Summary statistics for HYOU1 are from Koprulu et al.^12^, all other statistics were obtained from the OpenGWAS database^61^ and are listed in Supplemental table 4.

Coronary artery disease (CAD) was the most frequently genetically linked disease with a total of eight distinct protein targets that also changed during fasting (Fig. 7A-C), including four not related to lipid metabolism such as a reduction in leiomodin 1 (LMOD1) that has been previously suggested to be involved in smooth muscle cell differentiation^46^. Out of the latter, a link between hypoxia up-regulated 1 (HYOU1) and CAD has not been described. Briefly, the HYOU1-increasing T-allele (beta=0.20; p<2.8x10^-16^) of the shared genetic variant rs1177562 (MAF=40.3%; PP=95.1%; Fig. 7D) was associated with an increased risk for CAD (beta=0.03; p<2.9x10^-7^) and we estimated a two-fold increased risk per 1 s.d. increase in genetically predicted plasma protein levels (odds ratio: 2.02, 95%-CI: 1.54-2.64; p<3.0x10^-7^) after correction for case/control imbalance. We identified evidence for this genetic signal to also be linked to an adverse cardiovascular risk profile, including higher adiposity, fat mass, liver enzyme levels, and lower HDL-cholesterol (Fig. 7D and Supplemental Tab. 4). HYOU1 is highly expressed in tissues and cell-types rich in endoplasmic reticulum such as liver and pancreas but also plasma cells, and dendritic cells in adipose tissue^22^, where it contributes to correct folding and secretion of proteins. It belongs to the heat shock protein 70 family and accumulates in the endoplasmic reticulum under hypoxic conditions^47^, such as during reduced cardiac blood flow due to atherosclerotic plaques causing ischemia and subsequently CAD. While we provide robust genetic evidence that elevated levels of HYOU1 increase (life-long) CAD risk and demonstrated that prolonged fasting potentially compensates by decreasing plasma levels, the exact mechanism remains to be established.

## Discussion

Lack of food has been the default situation throughout human evolution, and our bodies are the result of a selection process for high metabolic flexibility to survive long periods without it. Today, fasting is practiced by billions of people world-wide for health or religious purposes, but how humans adapt to prolonged food deprivation beyond changes in fuel utilization and how this may translate into beneficial or adverse health effects beyond weight loss is largely unknown. Here, we demonstrate that more than 1000 plasma proteins show differential abundance following a seven-day water-only fast, with most potentially health relevant changes not occurring until after three days of consecutive fasting. We identify distinct temporal patterns in the plasma proteome as a multi-organ response to fasting, that, among others, highlights a progressive loss of the ECM at various sites possibly accounting for the loss in lean mass. We exemplify the translational potential of our results by identifying putative causal mediators of the health benefits of fasting, like SWAP70 for RA or HYOU1 for heart disease, through integration of human genetic evidence. Our results provide the opportunity to systematically identify the potential health benefits from fasting and translate this knowledge into putative interventions, including for patients who cannot adhere to prolonged fasting schemes or fasting mimicking diets^29^.

The lag in the onset of a strong fasting signature in the proteome compared to an almost continuous effect on weight loss uniquely allowed us to dissect effects that differ between fasting and weight loss as well as fasting of different durations. Our results suggest that profound non-metabolic adaptions among healthy volunteers, possibly providing a positive health impact over and above weight loss, do not happen before two, or even three days of prolonged food deprivation. Findings in line with meta-analyses^17,18^ and the general conception in the field^6^, that short-term (≤48h), intermittent fasting schemes are not superior to matched calorie restrictions to reduce markers of cardiometabolic risk. Large, prospective clinical trials are needed to proof our genetic predictions, but few studies already reported a general improvement in cardiometabolic health during week(s)-long fasting schemes with minimum calorie intake^48^. This is of particular relevance, since most proteins returned to baseline levels after participants resumed eating, suggesting that positive health effects might only be achieved through sustained manipulation of relevant targets.

Our temporal dissection of plasma proteomic profiles provided insights beyond the merely energy-centric view on fasting and starvation. We observed numerous non-metabolic and even transient findings. For example, a strong decline in EPHA1 may indicate altered epithelial cell motility^25^. The transient increase in the abundance of members of the complement cascade around day 2 and 3, which are usually perceived as the most challenging days by volunteers, possibly due to severe metabolic stress as glycogen pools become depleted^19^, may hint towards shifts or reorganisation in the innate immune system. Similar phenomena in the adaptive immune system might explain the transient decrease in many intracellular proteins that returned to baseline still during fasting. It remains to be established how persistent these changes are, and whether they causally contribute to the lower inflammatory burden reported for prolonged fasting^3^.

Although our study is distinguished by its comprehensive profiling of the proteomic response to fasting and the integration of human genetic evidence, it does have limitations. Firstly, the sample size (n=12) and the homogenous composition of our volunteers restricts the generalizability of our results as did a missing control group. Larger studies in diverse populations, including patients in severe states of starvation, like cachexia, are needed to corroborate our findings. However, our findings show some level of concordance with findings for selected targets during short-term fasting in ethnically diverse groups, like Ramadan fasting^49^. Secondly, our predicted ‘compensating’ effects of fasting on dozens of potentially disease-causing proteins should be considered exploratory. The frequency and intensity of fasting schemes required to translate this into preventive or symptom mitigating effects is unclear, and our results should not be interpreted as fasting being a sole solution to the disease examples shown. Thirdly, while the plasma proteome can be seen as convergence of a multi-organ response, the coverage of our proteomics platform is still limited and other proteomics and more generally ‘omics techniques, also targeting other tissues, possibly even brain, will provide complementary insights. Fourthly, while we see a segregation of fasting and weight loss associated proteomic profiles, longer term studies are needed to fully characterize the proteomic response of weight loss^50^. Fifthly, while we saw only weak evidence for a severe catabolic state driving proteolysis, proteins that changed only late (e.g., cluster 7) might well be a consequence of such unspecific systemic effects. Finally, the response to fasting may well have a diurnal rhythm missed by our sampling scheme, and longer time intervals with more frequent sampling will even further enhance our understanding of the dynamics underlying the adaptions to one of the greatest evolutionary challenges.

In summary, we demonstrated a highly conserved, multi-organ response in humans during prolonged fasting via the plasma proteome that extends beyond an energy-centric view and provides tangible insights for clinical translation. Our results will provide an important reference for the interpretation of future fasting and weight loss studies of different durations, including intermittent schemes.

## Methods

### Study design

We studied twelve apparently healthy non-smoking participants of mainly (all but one) white-European ancestry (exclusion criteria: any known disease and percent body fat below 15% for females and 12% for males). During a stringent seven-day fasting regimen only water intake was allowed. We sampled fasting blood specimens in the morning two days before study onset, each day under study, as well as after three days of *ad libitum* food intake. Blood samples were drawn from the antecubital vein and immediately placed on ice. Plasma EDTA samples (VACUETTE® EDTA-K2; Greiner bio-one, Kremsmüster, Austria) were centrifuged (3500 g at 4 ºC for 10 minute), transferred to Protein LoBind Eppendorf tubes (Eppendorf, Hamburg, Germany) and immediately frozen on dry-ice and stored at -80 °C. Weight of study participants was recorded every morning at an accuracy of 0.1 kg (Seca 877, Seca Gmbh, Hamburg, Germany).

All participants gave their written informed consent, and the study was approved by the local ethic committee (Norwegian School of Sport Sciences, #15-220817).

### Clinical chemistry assays and body composition

Plasma insulin, free fatty acids, and glucose were analysed as described^51^. Plasma 3-hydroxybutyrate was measured enzymatically, based on the oxidation of 3-hydroxybutyrate to acetoacetate by the enzyme 3-hydroxybutyrate dehydrogenase (Randox Laboratories Ltd., Crumlin, United Kingdom) using the Cobas C111 system (Roche Diagnostics, Rotkreuz, Switzerland). Blood lipid, liver enzyme, and thyroid hormone parameters were determined with the use of direct and enzymatic assay (Roche Diagnostics, Rotkreuz, Switzerland) and analysed on a Roche Cobas C111 Clinical Chemistry Analyzer (Roche Diagnostics, GmbH, Rotkreuz, Switzerland). Nitrogen in urine was measured with the Kjeldahl method^52^ based on 24h urine collections.

Body composition was determined by dual-energy X-ray absorptiometry (DXA) after overnight fasting according to recommendation (Lunar iDXA, GE Healthcare, Madison, USA) and analysed using enCORE software (v18; GE Healthcare, Madison, USA). Two DXA scans were performed before the fasting period started (on day -4 and -1), after 7 days of fasting, and three days after finishing the fasting period (day 10). The mean of the two DXA determinations before the fasting was used to describe body composition at baseline.

### Proteomics measurements

We measured plasma levels of a total of 2923 protein targets using 2941 assays distributed across eight panels based on the Olink® 3072 Explore platform from all plasma samples obtained. Assay details have been described in detail^53,54^. Briefly, proteins are targeted by two separate antibodies labelled with complementary single stranded oligonucleotides (proximity extension assays, PEA). Upon binding of antibody pairs with complementary oligonucleotides, these hybridize and can be quantified using next generation sequencing (NGS) to generate normalized protein expression (NPX) units, reported on a log2 scale. Olink’s quality control and normalisation procedures involve incubation, extension, and amplification controls, as well as negative, plate and sample controls. Values are generated by normalization to the extension control and further normalisation to the plate controls. Almost 80% (n=2334) of the protein targets had >75% values above the detection limit of the assay. We included all reported NPX values, even those below LOD, to ensure a full data matrix, a procedure very similar to random imputation of small values. We excluded measurements for two participants from three and one panels, respectively, due to outlying protein quantities based on recommendations from Olink. To facilitate interpretability of effect estimates, we scaled all NPX values for each protein by the mean and standard deviation prior fasting. This allows to interpret changes in terms of standard deviation units even in the absence of quantitative measures.

### Statistical analysis

We used linear mixed models to test for a significant change compared to baseline at any timepoint. Each protein was tested separately and with timepoint as the main fixed effect and participant as random effect. This analyses were implemented with the R package *lmerTest*^55^ and we subsequently used the *aov()* function to obtain a global p-value for any change during the study period. To test for potential sex-differential effects we additionally included a time * sex interaction term in the model. We again used mixed effect linear regression models to test for associations between weight as well as plasma levels of 3-hydroxybutyrate, insulin, glucose, total triglycerides, and free fatty acids as exposure and plasma proteins as outcomes modelling time and participant as random effects. If not otherwise stated, we used the Benjamini-Hochberg procedure to control the false discovery rate (q-value) at 5%.

We performed pathway enrichment analysis using the R package gprofiler2 (v0.2.1)^56^ restricting to KEGG and REACTOME database to maintain specificity. We used all protein coding genes covered by the Olink Explore platform as a background and tested for enrichment of 1) all significantly altered proteins, and 2) restricting to proteins belonging to temporal clusters. We again used the Benjamin-Hochberg procedure to account for multiple testing. For visualization purposes, we pruned redundant pathways iteratively, by first selecting the pathway with the largest coverage and subsequently deleting all pathways that contained at least halve of the genes. We repeated this procedure until no further pathways remained to be deleted. This means that pathway enrichment results presented in figures cover distinct set of genes/proteins. We further used the *pathview* (v1.34.0)^57^ R package to visualize selected pathways.

All statistical analysis were implemented in R v.4.1.0.

### Tissue and single-cell mapping

To understand the possible tissue origin of the changes in the plasma proteome upon fasting, we programmatically downloaded tissue- and cell-type specificity data from the Human Protein Atlas (HPA)^22^ for the Olink proteins in JSON format (on 30.12.2022).

Before joining HPA data with Olink data, we split Olink IDs corresponding to multiple proteins (protein complexes) into their components based on ENSEMBL gene IDs; and only the results with the lowest q-value were retained downstream if multiple Olink IDs targeted the same protein. Nine proteins (AKR7L, ANP32C, BTNL10, FHIP2A, HCG22, KIR2DL2, KIR2DS4, LILRA3, PNLIPRP2) assayed by Olink were not found on HPA, and NTproBNP was assigned to NPPB, leaving 2918 unique protein targets.

To determine if proteins that HPA reports as tissue- or cell-type specific were enriched among the fasting proteome, we performed a two-sided Fisher’s exact test for each tissue or cell-type, with the number of significant / non-significant and specific / non-specific proteins. We defined tissue- or cell-type specific as ‘enhanced’, ‘enriched’, or ‘group enriched’ according to Human Protein Atlas classification. Some proteins were specific to multiple tissues or cell-types.

### Clustering of temporal trajectories

To cluster protein targets with a similar behaviour throughout the study period, we used a recently proposed modelling approach for summary statistics that fits a Noise-Augmented von Mises–Fisher Mixture model (NAvMix)^58^. Briefly, instead of using individual level data, we used effect estimates and standard errors from the linear mixed models to obtain an average estimate with uncertainty for the change in protein levels. We subsequently scaled effect estimates using standard errors and subjected the matrix to the *navmix* function of the R *navmix* package (v.0.2.1) using default parameters. In contrast to most clustering methods that are based on Euclidean distances, NAvMix models effect directions to cluster associations using Mises-Fisher distributions that further includes a noise cluster. The output of the algorithm is a probability for each protein to belong to each of the proposed clusters. We first run NAvMix with a maximum number of 20 clusters and subsequently examined a plateau effect in the Bayesian Information criterion describing the fit for each of one to twenty cluster solutions. Since we observed a plateau effect at nine clusters, we rerun NAvMix with a fixed cluster number to obtain final assignments for each protein target. We used a hard clustering approach, assigning each protein to only one cluster.

### Genetic analysis

We downloaded genome-wide association statistics for plasma protein levels from Koprulu et al.^12^ to perform Bayesian fine-mapping in protein encoding regions (±500kb). We note, the provided statistics represent a meta-analysis of the discovery and replication set described by Koprulu *et al*. , doubling the sample size for discovery, and we hence sought to identify genetic signals de novo, including those not described by the authors. To this end, we first extracted association statistics for the relevant genomic region, excluding the extended MHC region (chr6:25.5-34.0Mb), and used the ‘sum of single effects’ model (SuSiE)^59^ to identify independent sets of genetic variants within each region, so-called credible sets, associated with plasma protein levels (cis protein quantitative trait loci – cis-pQTL). Briefly, SuSiE employs a Bayesian framework for variable selection in a multiple regression problem with the aim to identify sets of independent variants, each of which likely contain the true causally underlying genetic variant. We implemented the workflow using the R package susieR (v.0.11.92) and used the *susie_rss()* function to work with summary statistics, instead of individual level data. We used default parameters except for that we iterated L, the maximum number of credible sets, from two to ten, and only took the results for L with the largest set of independent (pairwise r^2^<0.25) forward, since SuSiE sometimes tended to produce correlated credible sets. We identified a total of 1491 credible sets for a total of 679 protein targets significantly changed during fasting.

We next systematically tested whether any of the identified cis-pQTLs does also affect complex phenotypes and diseases by performing phenome-wide colocalization screens similar to our previous work^11,12^. Briefly, we first queried cis-pQTLs in the OpenGWAS data base and subsequently tested suggestive findings (p<10^-6^) for a shared genetic signal using statistical colocalization, a test that provides posterior probabilities for five hypotheses, among which we concentrated on ‘H4’, that the protein and the trait share a genetic signal at the protein encoding locus. We adopted the prior for a shared genetic signal p_12_ to 5x10^-6^ and used a recently developed extension, which employs fine-mapping before colocalization to account for multiple causal variants in the region^60^. We considered a shared signal, if PP for H4 was ≥80% and the shared signal was the lead cis-pQTL in a credible set or a strong proxy thereof (r^2^>0.8). We visualised cis-pQTL – protein – trait results into a bipartite graph using the *igraph* R package.

## Supplementary Material

Supplementary Materials

## Figures and Tables

**Figure:**
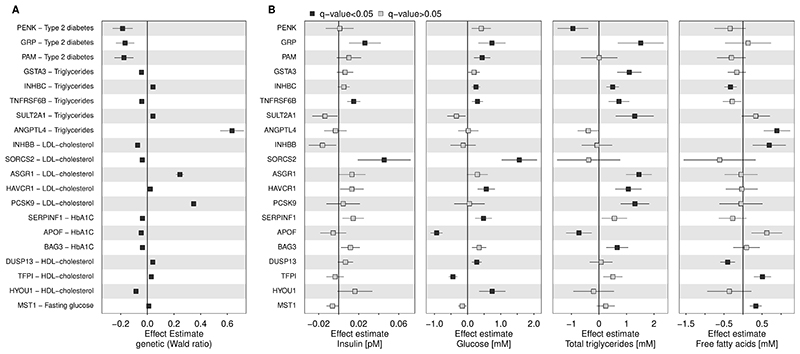


## Data Availability

GWAS summary statistics for proteins are available from an interactive webserver (https://omicscience.org/) and statistics for other outcomes have been obtained from the OpenGWAS data base (https://gwas.mrcieu.ac.uk/).

## Code availability

Associated code and scripts for the analysis will be made available upon publication.
