# Supplementary material for: Systemic Adaptions to Extreme Caloric Restrictions of Different Durations in Humans
Source: Nat Metab. Author manuscript; Available in PMC 2025 Jan 18. (PMC7617311; doi:10.1038/s42255-024-01008-9)
Supplement: Supplementary Materials [file EMS202181-supplement-Supplementary_Materials.pdf]

Systemic Adaptions to Extreme Caloric Restrictions of Different Durations in Humans

Maik Pietzner[1,2,3], Burulça Uluvar[1], Kristoffer J. Kolnes[4,5], Per B. Jeppesen[6], S. Victoria Frivold[7], Øyvind Skattebo[4], Egil I. Johansen[4], Bjørn S. Skålhegg[8], Jørgen F. P. Wojtaszewski[9], Anders J. Kolnes[10], Giles S. H. Yeo[11], Stephen O'Rahilly[11], Jørgen Jensen[4], Claudia Langenberg[1,2,3]

[1]Computational Medicine, Berlin Institute of Health at Charité – Universitätsmedizin Berlin, Berlin, Germany
[2]Precision Healthcare University Research Institute, Queen Mary University of London, London, UK
[3]MRC Epidemiology Unit, University of Cambridge, Cambridge, UK
[4]Department of Physical Performance, Norwegian School of Sport Sciences, Oslo, Norway
[5]Steno Diabetes Center Odense, Odense University Hospital, Odense, Denmark
[6]Department of Clinical Medicine, Aarhus University, Aarhus, Denmark
[7]Institute of Health and Society, Faculty of Medicine, University of Oslo, Oslo, Norway
[8]Department of Nutrition, Division for Molecular Nutrition, University of Oslo, Oslo, Norway
[9]August Krogh Section for Molecular Physiology, Department of Nutrition, Exercise and Sports, University of Copenhagen, Copenhagen, Denmark
[10]Section of Specialized Endocrinology, Department of Endocrinology, Oslo University Hospital, Oslo, Norway
[11]Metabolic Research Laboratory, Wellcome-MRC Institute of Metabolic Science, University of Cambridge School of Clinical Medicine, Cambridge CB2 0QQ, UK

Corresponding authors
Maik Pietzner, PhD (maik.pietzner@bih-charite.de)
Prof Claudia Langenberg (claudia.langenberg@qmul.ac.uk)

FIGURES

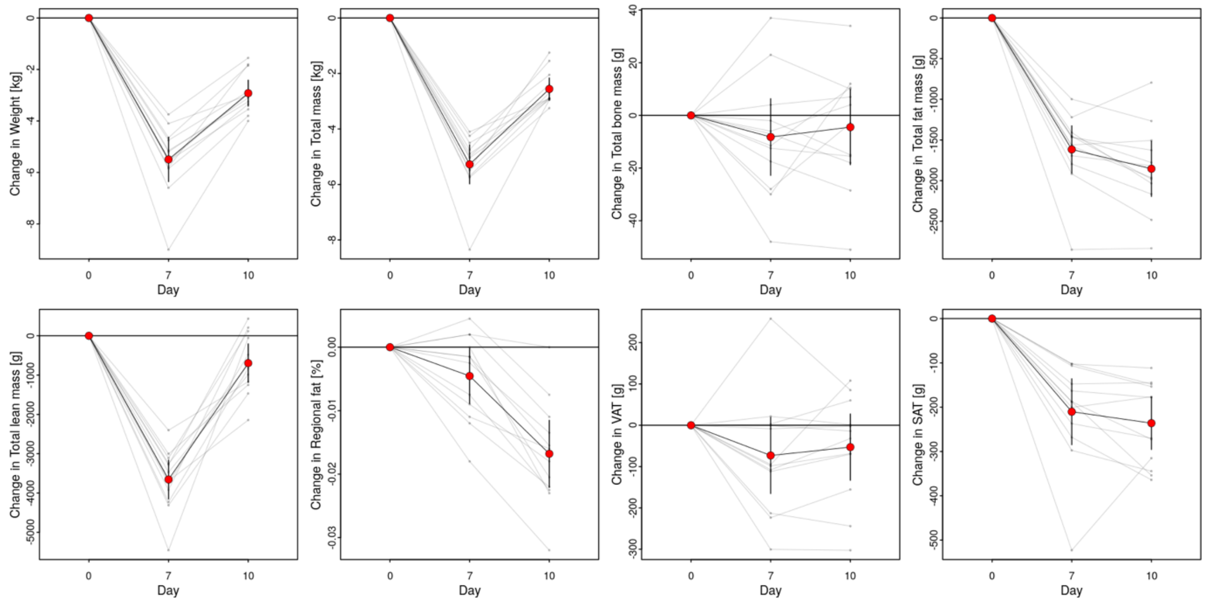

Supplementary Figure 1 Change in body composition as measured by dual-energy X-ray absorptiometry (DEXA). Each panel contains a separate measure and mean ± SEM are displayed for a change compared to baseline values. Corresponding association statistics can be found in Supplementary Tab. 1. SAT = subcutaneous adipose tissue; VAT = visceral adipose tissue

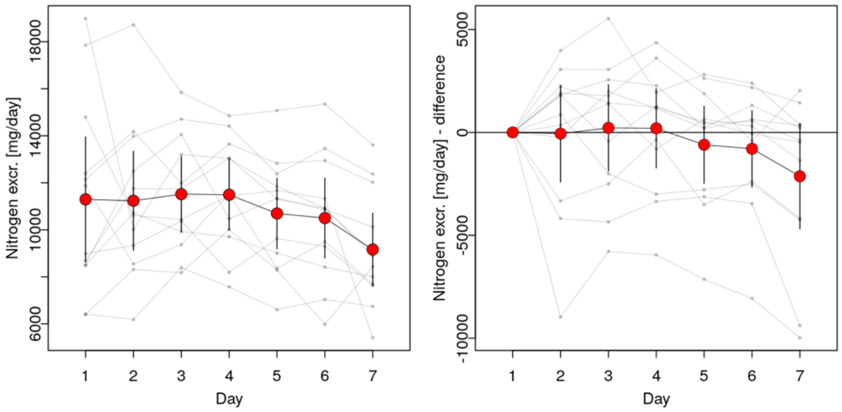

Supplementary Figure 2 Change in urinary nitrogen excretion during the time course of the study. Each panel contains a separate measure and mean ± SEM are displayed for a change compared to baseline values. Corresponding association statistics can be found in Supplementary Tab. 1.

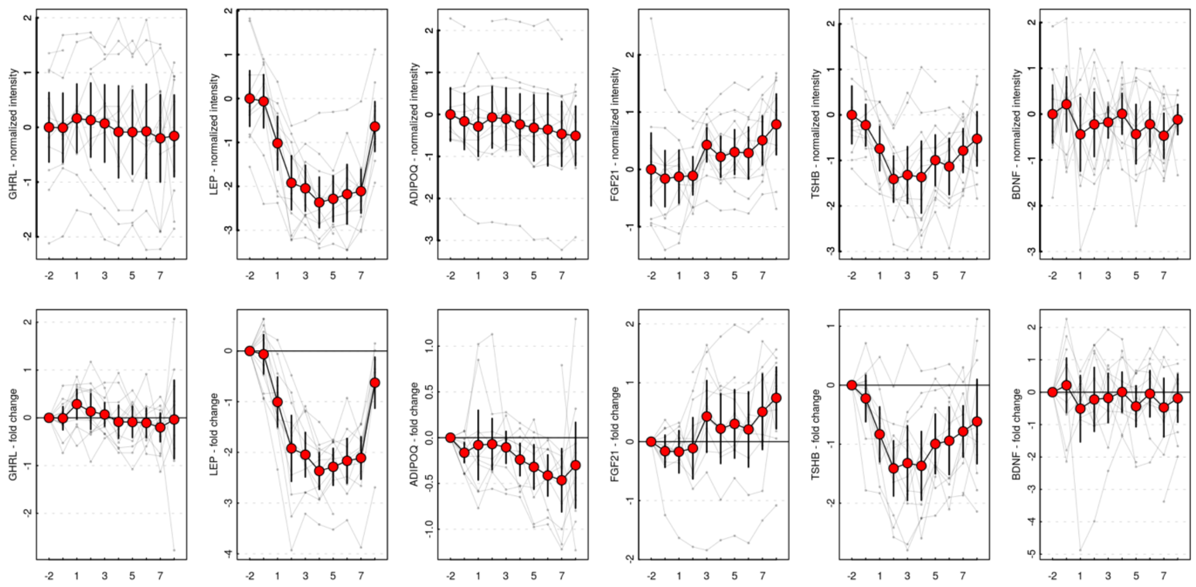

Supplementary Figure 3 Individual time courses of selected protein candidates. The upper panel displays mean ± SEM in original units for each time point of the study, whereas the lower panel displays mean ± SEM for change compared to baseline values. Thin grey lines indicate individual participants.

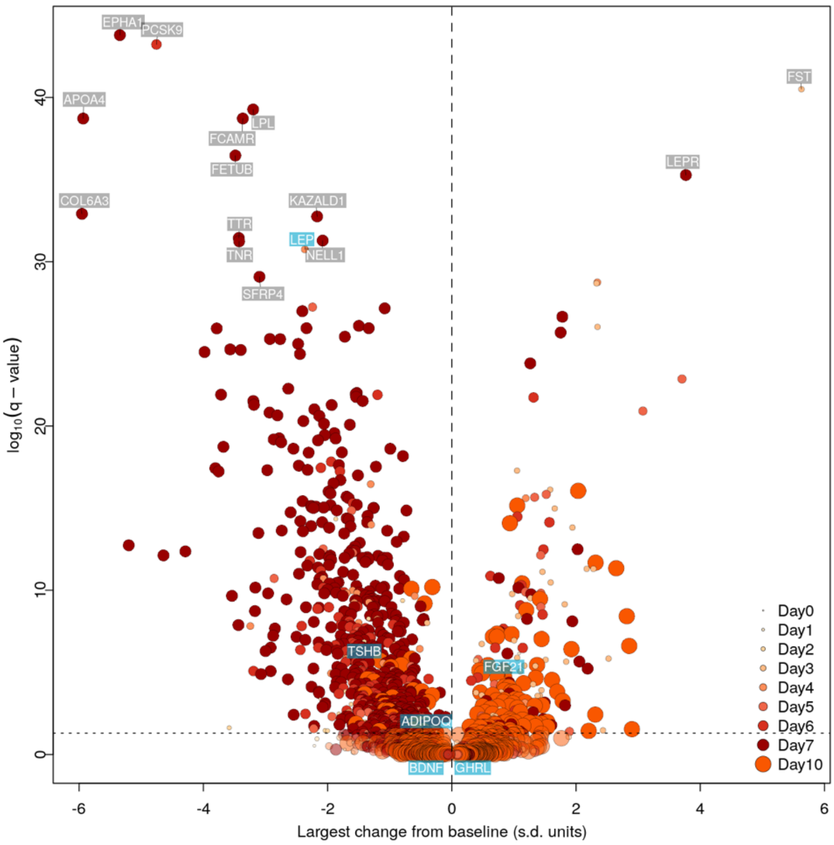

Supplementary Figure 4 Volcano plot of protein changes. The y-axis displays corrected p-values from mixed effect linear regression models for a time effect, whereas the x-axis displays the largest extend proteins changed during the study. The size of the dot indicates at which timepoint the largest average change was observed.

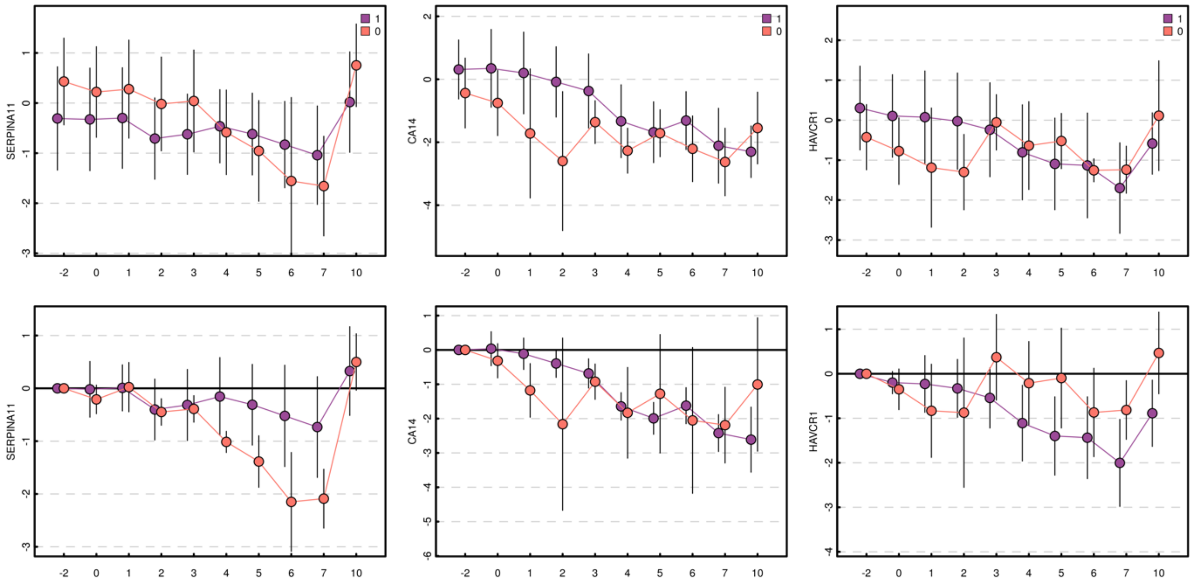

Supplementary Figure 5 Proteins with a sex-differential response during the study period. Sex-specific mean ± SEM values are shown for three proteins that showed significant evidence (q-value<0.05) for sex-differential effects. 1 = men; 0 = women. The upper panel displays original values, whereas the lower panel displays changes from baseline.

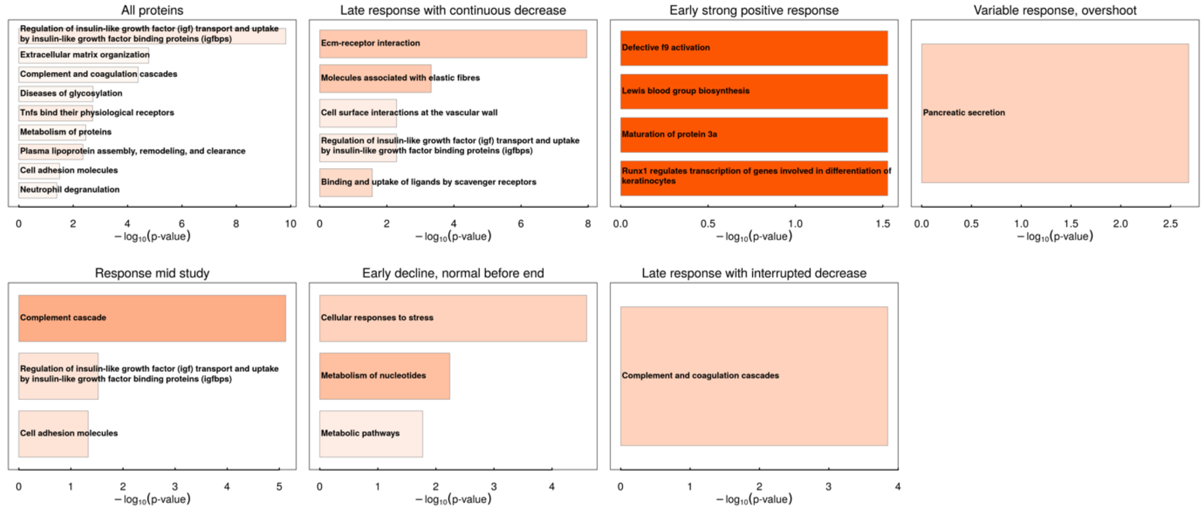

Supplementary Figure 6 Results from pathway enrichment analysis. The first box refers to results using all significantly altered proteins, whereas all remaining refer to one of the clusters of proteins shown in main figure 2. Each box displays the p-value (x-axis) and fold enrichment (colour intensity) for distinct set of pathways.

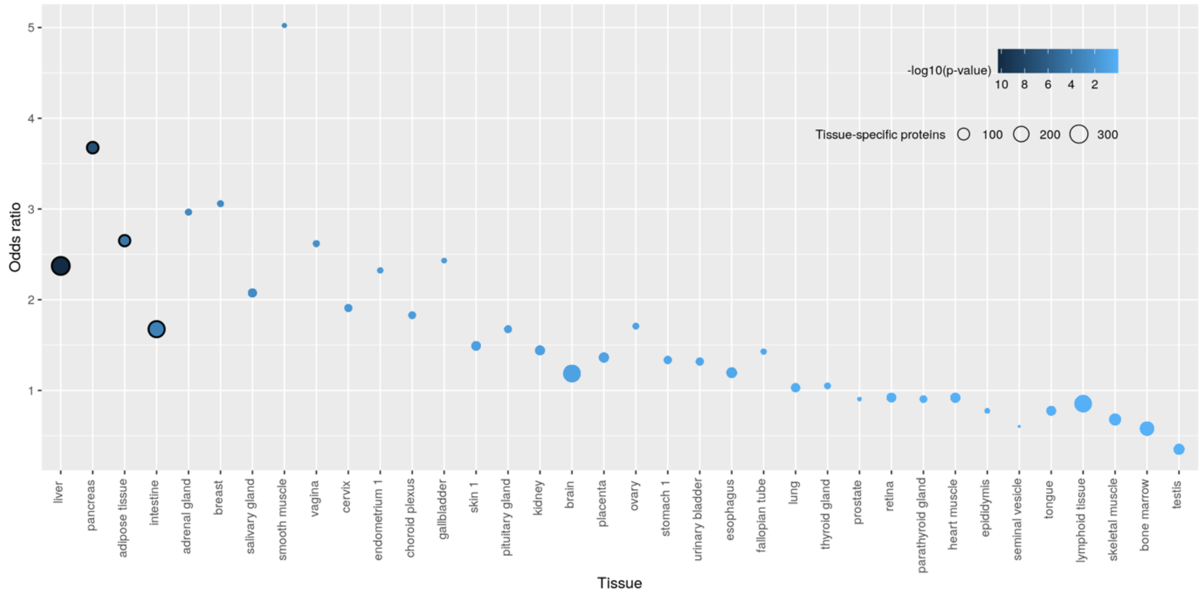

Supplementary Figure 7 Tissue enrichment of proteins altered during fasting. Plot displays results of Fisher's exact tests for the enrichment of proteins altered during fasting among tissue-specific proteins, according to Human Protein Atlas. The y-axis shows the odds ratio estimate, the x-axis is ordered by –log10(p-value). The sizes of the dots show the number of proteins that are both tissue-specific and their plasma levels change during fasting, and they have a black border if the Bonferroni-adjusted Fisher's p-value for 36 tissues is below 0.05.

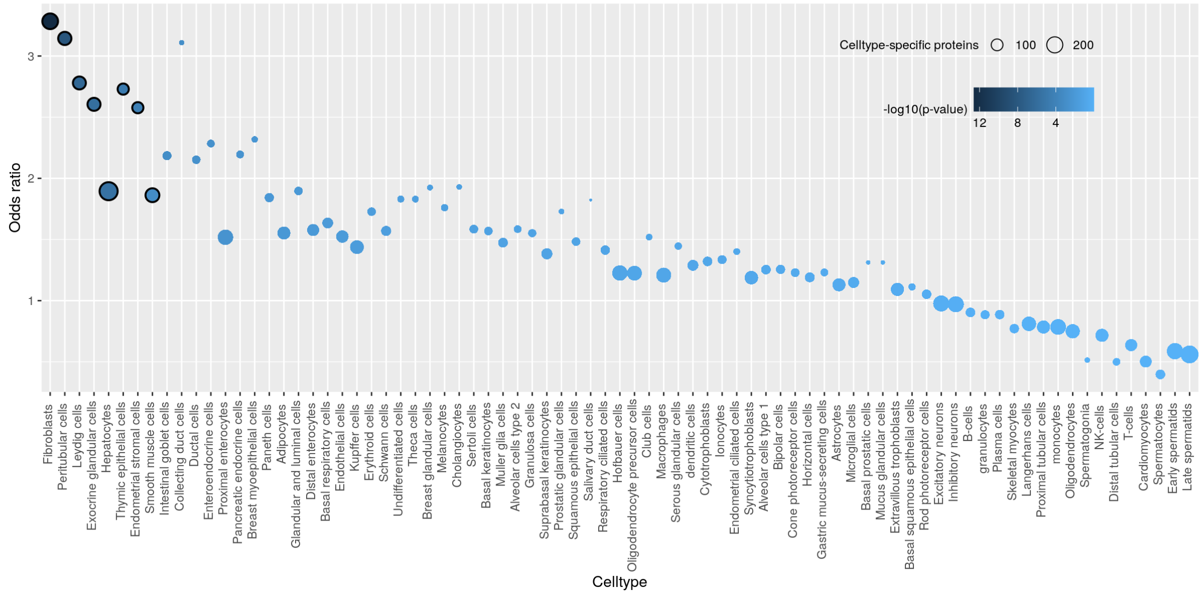

Supplementary Figure 8 Cell-type enrichment of proteins altered during fasting. Same as Supplementary Fig. 6, but for celltype-specific proteins according to Human Protein Atlas. Dots have a black border if the Bonferroni-adjusted Fisher's p-value for 79 cell types is below 0.05.

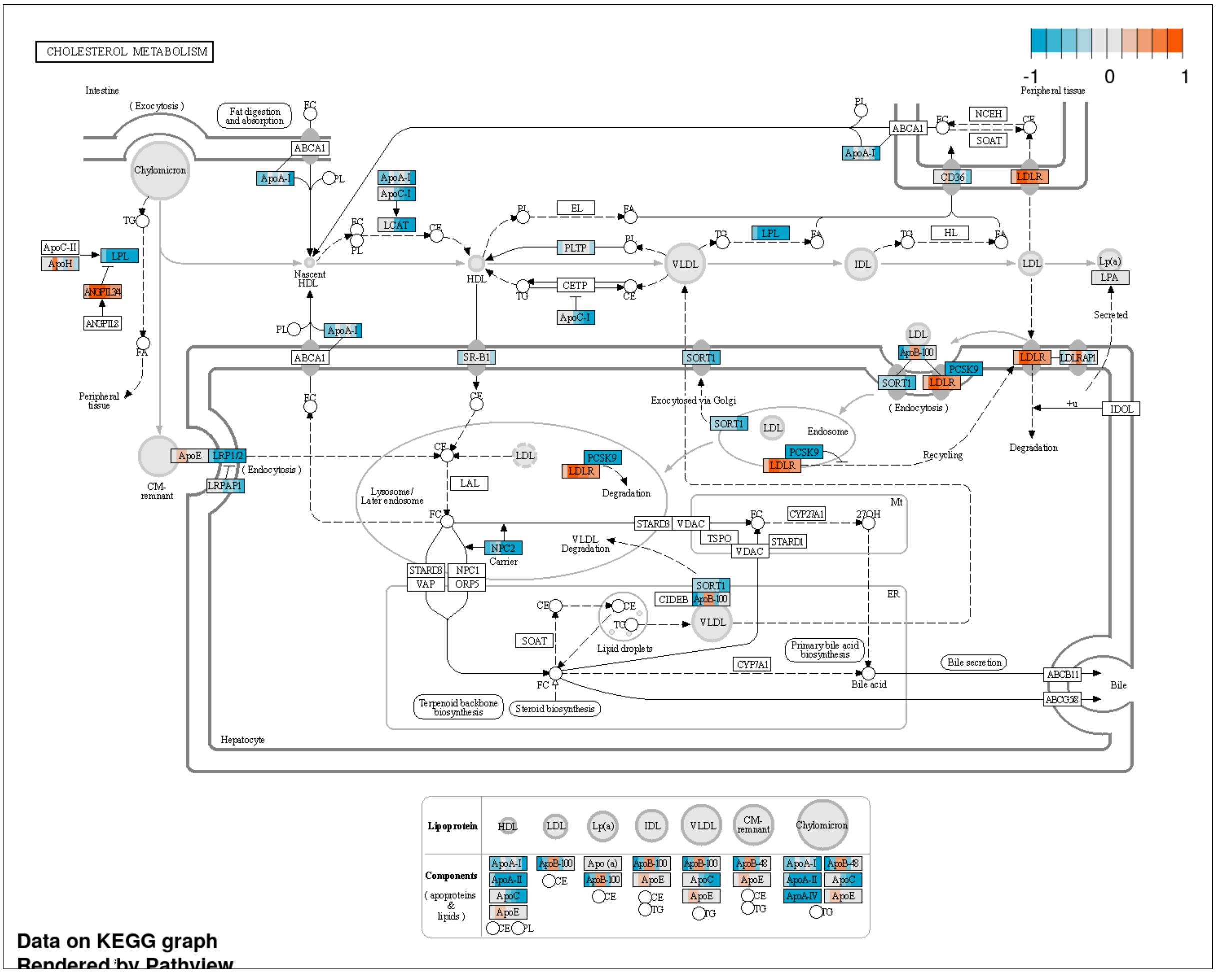

Supplementary Figure 9. Changes in proteins belonging involved in cholesterol metabolism during the study period. Each protein measured in the current study is coloured according to the trajectory during fasting. The colour gradient is based on effect estimates from linear mixed models and has been restricted to -1 and 1 to enhance visualisation.

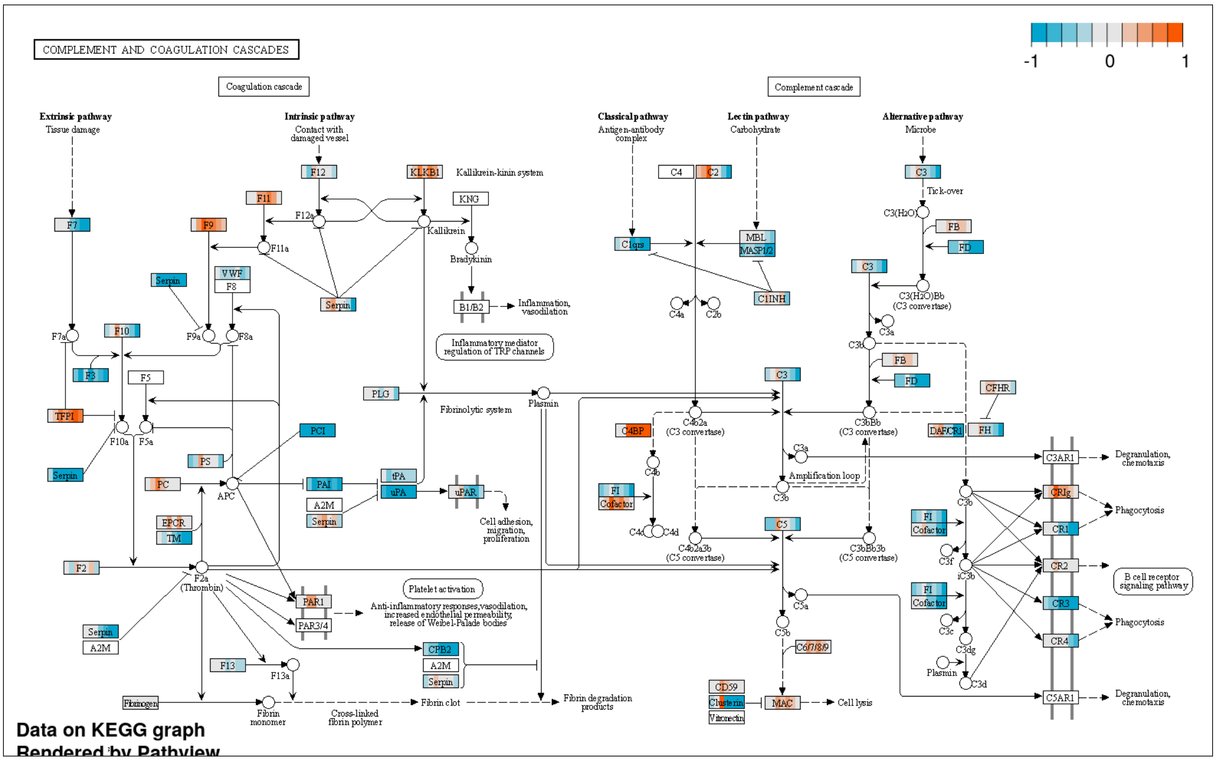

Supplementary Figure 10. Changes in the complement and coagulation cascade during the study period. Each protein measured in the current study is coloured according to the trajectory during fasting. The colour gradient is based on effect estimates from linear mixed models and has been restricted to -1 and 1 to enhance visualisation.
